# Examining how unemployment, inflation and their related aspects affected economic growth in Palestine: The period from 1991 to 2020

**DOI:** 10.1016/j.heliyon.2023.e21081

**Published:** 2023-10-20

**Authors:** Alaa Razia, Mostafa Omarya, Bahaa Razia, Bahaa Awwad, Abdullah Ruzieh

**Affiliations:** a An-Najah National University, Islamic Banking Department, Nablus, Palestine; b An-Najah National University, Nablus, Palestine; c Palestine Technical University – Kadoorie, Faculty of Business and Economics, Tulkarem, Palestine

**Keywords:** Inflation, Economic development, Unemployment and ARDL model

## Abstract

This study aims to focus on identifying how unemployment and inflation and their related aspects affect Palestinian economic growth. The study employed the Autoregressive Distributed Lag (ARDL) model employing annual time series data for estimate and result analysis. The ARDL model was estimated in both long and short run as follows; on one hand, the results showed that there is a potential negative impact of aspects-related unemployment on economic growth in Palestine over the long-term. On the other hand, over the long term also, it can be noticed that there is a positive impact of aspects-related inflation on economic growth in the state of Palestine. In the short term, a significant negative effect of unemployment (UN) was discovered on economic growth, while there was no effect of inflation (INF), despite being positive. The error-correction model (ECM) has been adopted in this model, and the results revealed that the ECM co-efficient [ECM (−1)] was positive and equaled to (0.0183) and therefore was not significant. Based on the results obtained, the study recommended that there a need to stabilize inflation through effective price control mechanisms that can enhance macroeconomic conditions, increase investor confidence, stimulate economic growth, and work to create opportunities for the full use of human resources by stimulating self-employment to engage in entrepreneurial activities according to the qualifications of graduates. Finally, it equips population, especially youth, with relevant skills to increase productivity and reduce unemployment.

## 1 Introduction

One of the most significant economic difficulties within any thriving economy is the challenges that face bridging the gaps between unemployment, inflation and economic development. It is hard to control high levels of unemployment and inflation at once in some countries due to the fact that these are sources of concern particularly in Palestine which is a country with human resources that has been ruled by occupation since 1948 [1]. These are major indicators of the general economy, which also play a key role in determining growth and development on a large scale. Economic growth is associated with a potential increase in the values of the market which are modified for inflation for numerous types of services and goods that are produced by an economy over a certain period of time. As a proportion of the possible increase in overall production and development, it is traditionally metric. In particular, unemployment means difficulty in finding any work for those prepared, actively searching for work opportunities and capable of acquiring it for a wage [2]. It is also vital to note that unemployed people need to be an active person in seeking remunerative work. Hence, Inflation, on the other hand, is an increase in the prices and their related aspects in terms of services, goods and products in a certain country, in addition to a potential decrease in the value of the currency used in that country [3].

In terms of the relationship between inflation and its related aspects and its possible impact on growth including employment, wages, investment, wealth, income distribution, policy and social conditions, inflation has a significant effect that can be positive or negative on almost every aspect of the state. This is because the negative measures of both unemployment and inflation are greatly related to economic stagnation, however, luckily, Palestine is one of many countries that has the lowest rates of inflation which means that inflation and its related aspects have a low impact on the economy [4]. Given that the Palestinian State does not have a central bank, carrying out and formulating policies related to monetary and fiscal, can be considered insufficient. For this reason, it is difficult to change or control the current used in the country since this current belongs to another country. In this context, the use of policies with an infrastructure which is different from traditional policy needs to be considered. An imbalance in the Palestinian labor market includes a mismatch of wages between workers, this is due to multiple reasons including, long-term unemployment, lack of jobs and interruptions of forced employment [5]. In a direct relationship, the unemployment rate is considered to be a mission for the development of housing [6].

Numerous research concentrating on different economies have shown the connection between unemployment, economic growth, and inflation. The idea for this connection was first developed by Ref. [7]. This study stated that there is an inverse and opposite link between unemployment and economic growth. Okun's Law indicates that any rise in economic growth causes the unemployment rate to fall [8]. modelled the link between unemployment and inflation, where a rise in inflation equals a fall in unemployment, leading to the creation of the phrase “Phillips curve”. Because they disregarded the detrimental effects of inflation's long-term effects on the economy, it is therefore possible to carry out a demand policy to effectively attain inflationary growth. Even though the short-term impact is minimal, it is preferable to long-term harm. As a result, the impact of economic growth on inflation is substantially smaller compared with the overall impact of the increment rate of liquidity on inflation [9].

A variety of elements influencing the criterion have been presented by several research on economic growth, including the tracking of capital, employment, and technical advancements inside the nation. These objectives are set in terms of quantity and quality, which is distinct from the internal development process, which makes a substantial contribution to attaining overall economic development. Additionally, this bolsters Fisher's political decision-makers who concur that inflation generates more severe issues than unemployment. In fact, politicians and economists agree that reducing inflation is the first step toward achieving economic growth and infrastructure development. Additionally, preserving low inflation rates improves social welfare and raises citizens' quality of living.

Along with the independence of the local Central Bank and public confidence, adopting numerous policies, monetary rules and instruments for aspects related to fiscal and monetary is considered one of the crucial requirements that assist in managing and controlling the inflation rate and its potential impact on economic growth. The adoption of a suitable budget for the purposes of controlling inflation contributes to monetary policy's transparency and stability, which are other points worth mentioning. As a result, it must be guaranteed that the Central Bank will uphold its duty. Therefore, the connection between the policy and the Central Bank must be reciprocal [10].

It is noteworthy that the performance of Palestine has been stable since 1991 due to Israel's occupation of the Palestinian Territories and the merging of its economy with Palestine's economy from 1967 to 1993. However, the growth process in Palestine began in 1995, when the growth of the Palestinian economy was proceeding in a slow upward manner. The growth rate in 1994 reached about 2.43, reaching 7.1 % by the end of 2021 [4]. It confirms the vitality of the Palestinian economy despite the obstacles imposed on it. In the early years of 1991, Palestine was facing many problems and political crises that affected the unemployment rate, an unemployment rate of 10.2 %, but in 2000, the unemployment rate doubled to 22 % due to the second intifada, which caused a major economic recession. The unemployment rate continued to increase, due to crises and wars, it reached 26.2 %. Inflation reached about 5.23 at the beginning of 1991, but it decreased significantly over the years, reaching in 2016 (−0.21) [11]. The low level of inflation and the varying effect of this affected economic growth [12].

The aims of this research are the following: to examine how inflation and unemployment and their related aspects affect economic growth, particularly in Palestine. This leads to highlighting the quality of the relationship between unemployment and inflation on economic growth in the Palestinian economy. It also assists in proposing some basic policies on unemployment and inflation in growth for decision-makers within the Palestinian economy. This paper presents thirty years of data starting from 1991 to 2021 in order to examine the potential impact of unemployment issues and inflation rates on the country's growth. The “Cobb-Douglas Production Function” is the economic model employed in this study, with economic growth serving as the dependent variable and unemployment and inflation serving as the independent variables. In the econometric model, the researchers used the “Augmented Dickey-Fuller (ADF) test, and the Phillips Perron (P·P) test to measure the root of the unit.

## 2 Literature review

[13] employed time series data that has been obtained from the Central Bank of Nigeria between the time period of 1970–2010. Based on their studies, they found that the impact of inflation on the economic growth and market development of Nigeria is considered evident. The Unit Root Test and Granger's Test for Causality were also employed in order to evaluate the possible relationship between inflation and its related aspects and financial growth. This is related to the findings of the Granger Causality Test which revealed that there is a strong relationship between the flow in one direction from GDP to inflation. The Unit Root Test findings demonstrated that all factors, variables and rates were constant at the initial delay. In a comparable context [14], concurred with the earlier study regarding the identification of the impact of aspects linked with unemployment and inflation on Jordan's GDP. Furthermore, the data were gathered from the World Bank database between 2000 and 2010 and included time series data. To calculate the connection between the independent and dependent variables, the researchers employed the linear regression method. As a result, GDP will increase by 1% when the inflation rate increases by 0.906%. In contrast, GDP grows by 1% when unemployment falls by 0.697%. The study's conclusions showed that there was a significant positive correlation in addition to an inverse relationship between GDP and inflation and their impact on economic growth.

[15] examined the relationship between numerous economic aspects in Nigeria and clarified the link between these aspects including unemployment, inflation, and economic growth between the years 1987–2012. This relationship was carefully examined using secondary data. The ordinary least squares approach was the research methodology. The findings show that over time, inflation and unemployment will be able to have an opposite possible impact on Nigeria's economic growth, however, both interest rate and total public spending will have a substantial impact. The fact that inflation cannot be linked to pressure on aggregate demand but rather to problems with the supply chain of raw materials from domestic and international warehouses in 1996 and 2012 is one of the potential defenses for the negative effects of inflation on the price level. This study used ARDL to examine the short-term and long-term impacts of unemployment and inflation on economic growth. The model estimate shows a decline in inflation and unemployment in economic growth over the long run. This results in showing a large and adverse influence of both inflation and unemployment and their aspects and how they link with economic growth.

Statics and metrics of unemployment and inflation need to be carefully considered when examining the health of any economy. This is related to the previous study (Yelow, 2015) on how unemployment and inflation affected Sri Lanka's economic growth from 1990 to 2016. In this study, annual time series data from the World Bank Development Indicators were used, and the flexibility of long and short-run variables was investigated using co-integration. The stability features of time series variables were measured using the Augmented Dickey-Fuller Test [3]. The link between inflation, unemployment, and economic growth's long-run co-integration is limited by an autoregressive test method (ARDL) that validates the results of ARDL tests. According to the estimated test results, GDP growth and labor force participation have a strong inverse association, whereas inflation and GDP growth are greatly connected and have a significant positive relationship over time [16]. Analysis of the impact of inflation, population, unemployment, precipitation, and temperature on Saudi Arabia is greatly related to economic growth. It received annual time series datasets from the World Bank to examine the aspects that affect economic growth, and also from the Center for Meteorology and Environmental Protection, Kingdom of Saudi Arabia, spanning the years 1990–2019. The long-term relationship between all possible factors and variables was investigated using the autoregressive distributed technique (ARDL). The long-run ARDL model underwent multiple sequence analysis experiments to confirm its reliability. The study came to a number of conclusions, but the most significant one is that the Kingdom of Saudi Arabia is still able to experience higher economic growth levels without adequately identifying the issue of unemployment. This is because it was discovered that both variables are extremely important, but using the ARDL temporal model, effectively in a negative way. The short-term impacts of population expansion and the increase of prices in services and goods on economic growth vary, as they do in most other nations, but the long-term consequences have been found to have a positive impact.

[17] argued in their quantitative study, the potential effects of inflation and economic growth on aspects related to unemployment in Indonesia. The researchers selected time-series data analysis for 34 provinces in Indonesia over the period 2016–2018. The secondary data used in this study were sourced from Statistics Iceland. The study used Eviews 10 software to create a multiple linear regression test with a random effect model. These studies' findings demonstrated that Indonesia's unemployment is significantly and negatively impacted by both inflation and economic growth. The F-Test and *t*-Test indicate that neither economic growth nor inflation in Indonesia has a substantial impact on unemployment, either independently or together [18]. referred to the knowledge of the possible impact of inflation and unemployment on economic growth in Pakistan. Relevant data were gathered between 1980 and 2018 and the “Ordinary Least Squares [OLS]” approach was employed with several analytical procedures to determine the acceptability of the data for the study. Because higher t-statistic values of the t-tab and sig-value are also significant, econometric findings suggest that the time series are stable. The ADF's huge error time guarantees long-term communication. Inflation and unemployment rates are far from the equilibrium value; this is according to the ECM data. Inflation and unemployment are not statistically significant, and neither is the general model, according to the results of the multiple linear regression models [19]. tried to determine how the Philippines' economic development will be affected by inflation, unemployment, and population increase between 1991 and 2020. The World Bank's open data were used to collect the data for this study, hence the following tests will be used by the researchers: Root Test Unit and the Co-integration. The findings of this study indicate that inflation has a positive impact on economic growth, as indicated by the normal least squares results. Meanwhile, factors that have a negative effect on economic growth include unemployment and population increase. The unit root test demonstrates that inflation moves in the opposite direction of unemployment, population growth, and economic growth.

The aforementioned pillars of scientific studies direct researchers to the research methodology and work methodology and refer to the results of those studies to achieve integration and build on efforts. Additionally, the researchers benefit from the results and tools used, because the current study is considered a continuation of previous studies that dealt with the issue of the impact of inflation and unemployment on economic growth. To achieve the greatest benefit from the findings of studies and reviews of previous studies, the researchers aim to identify how inflation and unemployment affect the Palestinian economy by comparing the results that we will reach with previous studies. This study examines the potential effects of unemployment and inflation on aspects of economic growth analytically. The following are the study's hypotheses, which have been developed by the researchers from 1991 to 2020.

• Unemployment has a positive effect on GDP.
• Inflation has a positive effect on GDP.

This study is also interested in focusing on several aspects, the first aspect highlights a theoretical proposition of how the variables of the production function (Cobb Douglas) interact with each other. The negative and positive impacts of these variables are imported by the Israeli occupation state, and the impact of Palestinian politicians on the economy is directly to solve these imported problems. Therefore, this study will address the problem of unemployment and inflation and work on providing proposals to benefit from it in alleviating unemployment and imported inflation, especially after the coronavirus pandemic. Thus, the research will assist in achieving the ambitions of citizens towards the Palestinian government and will discover the great role of restoring the national economy through the above-mentioned studies. Comments will be made on previous studies as follows.

a Aspects of agreement between previous studies:

Previous studies focused only on the issue of applying variables and aspects of inflation and unemployment to economic growth, as this study is consistent with the previous studies in terms of the research topic and focuses on applying its measures. This study also agreed with most studies in terms of methodology, as most studies used the descriptive analytical approach.

b. Aspects of difference between previous studies:

This study differs in terms of the method of statistical analysis with the majority of studies that used one root unit test, which is the Dickie Fuller, unlike the current study, which added the Byron Phelps Test and the diversity of statistical tests. This aims to identify the actual result on the ground, which concentrates on secondary data in Palestine thus the study does not coincide with any previous study. What distinguishes this study from others is that it is the most comprehensive study on identifying how inflation and unemployment influence economic growth in Palestine.

c Scientific gap in the study:

This is a unique study that focuses on examining long-term time series in connection to how unemployment and inflation affect Palestinian economic growth. The previous studies differed from the current study in that all the countries on which studies were made are fully independent and control their economy in an organized manner. Palestine is an occupied country, and thus this shows a real desire among researchers to shed light on the mechanism of dealing with imported problems and how to find solutions to alleviate poverty and unemployment because no study close to the study population was observed. Moreover, not all of the previous studies agree with their findings.

## 3 Methodology

This research identifies how inflation and unemployment affect economic growth and its related factors in Palestine. The experimental model of the study was determined as follows:

(1) lnRGDPt=α0+α1lnInft+α2lnUnt+εt

*RGDP* refers to the dependent variable that represents real GDP as determined by economic growth.

INF and UN are the two regressive indicators that show inflation and unemployment.

T refers to the time measurements.

α 0 *and* α *i* (*w*ℎ*ere i* = 1, 2, …, 4) represent the intercept and the parameters that need to be evaluated.

ε*t* Refers to the supposed independently distributed random error term. This includes a constant variance as well as zero mean.

The dependent variable adopted in this study is gross domestic product (GDP). This variable is used to measure economic growth and to explain the average yearly growth of GDP in percentage terms (See Refs. [9,20]. Inflation is controlled by the annual growth of the consumer price index and it is anticipated that it will have a detrimental effect on economic growth. This is because of greater uncertainty and decreased investment. Moreover, unemployment (UN) is regulated as the overall percentage of the total labour force who is able to work and actively seeking any field of work. As a country grows economically, unemployment is expected to decrease based on the inverse relationship which is developed by Okun's Law. Moreover, in this study, Annual time series data from 1991 to 2020 from secondary sources were used, and the Eviews 12 statistical tool was employed for the statistical analysis. The analysis contained thirty observations. The data in the series was generated mainly from the National Bureau of Statistics, Palestinian National Bureau of Statistics, and the World Bank's Economic Publications website. It is also worth noting that all other parameters are logarithmic, and thus these parameters are easy to interpret and analyse.

## 4 Estimation procedure

a Unit Root Test

[21] stated that despite changes in time, the constant series maintain their structure, ruling out the existence of a unit root. Data of Time series typically exhibit erratic walking patterns. Regression analysis might produce false results if non-static variables are used. As a result, before conducting regression analysis, chain stability must be addressed.

The Augmented Dickey-Fuller (ADF) Test, despite the fact that there are numerous unit root tests, was frequently employed in this study. The Dickey-Fuller Test is expanded upon by the ADF Test [22]. Adopting the Phillips and Perron Test which is formulated by Ref. [23]. There are three ways to run these two tests: statically, according to a policy, or not at all. These tests primarily aim to compare the Alternative Hypothesis, “There is no unit root,” with the Null Hypothesis, “The string has a unit root.” The t-statistic is compared to the critical value to determine the stability of a sequence. It is assumed that the string is fixed at the level if null is rejected. In any case, anyone can flag the string and try again if it is not fixed.

b The Autoregressive Distributed Lag (ARDL) Model

In order to examine the correlation between unemployment and inflation and how they affect economic growth. The study chose the ARDL estimating approach. Because they offer accurate and effective estimates, the ARDL technique is appropriate when analyzing strings of mixed order, I (0) and I (1) [24]. This strategy has the benefit of allowing for delays in both dependent and independent variables, which lowers the likelihood of occurrence.

To ascertain whether there is a co-integration and connection between all variables of unemployment and inflation, the Bounds Test can be used using the ARDL approach. The presence of a long-term relationship between the variables is demonstrated via co-integration. This connection is established with the help of the Bounds Test. But for performing the ARDL Bounds test, choosing the best lag length is essential. The ideal latency is determined using a number of metrics, including several criteria such as; the Akaike Information Criterion (AIC), Schwarz Information Criterion (SIC), Hannan-Quinn Information Criterion (HQ), and Final Prediction Error (FPE).

Finally, this study has conducted an ARDL limit test by examining the Null Hypothesis of no co-integration against the Alternative Hypothesis of co-integration. This test assists in determining if there is a long-term relationship between the variables included in this study. Converting (Eq (2)) into ARDL form becomes as follows:

(2) $$\Delta RGDPt=\alpha 0+\sum_{i=1}^{n}\alpha 1\Delta \mathrm{Ln}\left(RGDP\right)t-i+\sum_{i=1}^{n}\alpha 2\Delta \mathrm{Ln}\left(\mathrm{INF}\right)t-i+\sum_{i=1}^{n}\alpha 3\Delta \mathrm{Ln}\left(UN\right)t-i+\beta 1\;\mathrm{Ln}\left(GDP\right)t-1+\beta 2\;\mathrm{Ln}\left(\mathrm{INF}\right)t-1+\beta 3\;\mathrm{Ln}\left(UN\right)t-1+\mu 1t$$

Where:

α0 refers to the constant, α1- α3 refer to regression coefficients, and μ1-μ3 refer to white noise error terms.

RGDP refers to Real GDP.

INF revers to Inflation rate.

UN refers to the Unemployment rate.

c The Error-Correction Model (ECM)

The Error Correction Model (ECM) is a member of a group of data-intensive multiple time series models when the underlying variables exhibit cointegration, often known as a common random long-run trend. For calculating the immediate and long-term effects of one time series on another, ECMs are a practical theoretical method. The idea of error correction refers to how the short-run dynamics of a system are impacted by the last period's error, or departure from long-run equilibrium. ECMs directly estimate the rate at which the dependent variable returns to equilibrium after a change in other variables has occurred. Thus (Eq (3)), is used to construct an error-corrected model, resulting in the OLS least squares model as follows:

(3) ΔRGDP=α0+β1Ln(GDP)+β2Ln(INF)+β3Ln(UN)+σECM+μ1

Eq (3) demonstrates a model with an error correction term (ECT) in the first difference. The ECM displays how quickly the model corrects for any short-run and long-run disequilibrium.

## 5 Results and discussions

a Test for Unit Root

The variables were examined to see if they had a unit root or not. As a result, the four variables were examined using the Dickey-Fuller Extended Root Unit Tests, Phillips, and Peronne tests. The outcomes of this study are displayed in Table 1.

**Table 1:** The unit root test statistics for ADF and PP.

	L		1st diff	
V	ADF	PP	ADF	PP
ln(RGDP)	−1.523062	3.764888	−4.147631*	−2.608057*
Ln(UN)	−1.250292	−1.196503	−4.743734*	−4.847395*
In(INF)	−3.092912**	−3.010785**	-	-

*,**,***represent 1 %,5 % & 10 % significance levels, respectively.

Numerous econometrics techniques make use of constant variables that are maintained constant. The aforementioned findings demonstrate the null hypothesis which indicates that the proposed information is stable and constant, is accepted. However, the void hypothesis—which contends that the data are unstable if the test results show that LnRGNP (real GDP) is not steady—is rejected. It was determined that the test statistic was (−1.523062). This statistic's total value fell short of the peak values at the 1 %, 5 %, and 10 % levels. After one difference, the test statistic was calculated as (−4.147631). As a result, at all three levels, the absolute value of this statistic was maintained and was also attained above the necessary levels. Furthermore, the Phillips-Perwin (PP) Test which refers to fixing the outcome at the second difference for the same variable since the In (INF) variable is considered the only one whose hypothesis was greatly accepted without any variance, serves as evidence of this. Additionally, because it is difficult to perform the Johansen Statistical Co-Integration test, as a result, the automatic regression of the distributed delay was used in the test in order to determine the possible relationship between different aspects including inflation, unemployment and economic growth. This has been performed in both the long and short term.

b Optimal Lag Selection

In order to choose the most suitable lag for the proposed model, this study used the lag series selection criteria. This step was important because the use of ineffective time periods may lead to inaccurate estimation results. To avoid this problem, a survey of delay length criteria was introduced. In this study, the Schwartz information criterion (SIC), the Akaike Information Criterion (AIC), the Hannan-Quinn criteria (HQ), and the final prediction error (FPE) were used as delay length criteria. These criteria help evaluate the quality and suitability of different delay times. Given that the data is quarterly, the study found it appropriate to consider four delays for the model, which is supported by previous research. By examining, the results presented in Table 2, the (SIC) and (HQ) latency criteria consistently indicated that delay 1 is the optimal delay for use in the model, and therefore one of them had to be used, and the researchers chose the most common Schwartz Information Criterion (SIC) criterion.

c (ARDL) Model in the short run

**Table 2:** Selecting appropriate lag.

lag	FPE	AIC	HQ	SIC
0	.000025	−2.09393	−2.05336	−1.94766
1	4.1e-07	−6.19681	−6.03454*	−5.61175*
2	4.7e-07	−6.10287	−5.8189	−5.07902
3	4.9e-07*	−6.39376*	−5.98808	−4.93111
4	4.0e-07	−6.36167	−6.03429	−4.66023

It must be ensured that the proposed model examines the possible balance and relationship among different variables in both the long and short term. For this reason, the Palestinian economy model was formulated and redeveloped prior to examining the possible relationship and its balance between different variables of the automatic regression model of distributed lag. As a result, these approaches are adopted in this study as seen in the following equation (Eq 2):

Ln(GDP)=Ln(INF)+Ln(UN)+μt

Table 3 will provide the findings of the error correction model, and the ideal number of delays for the model variables, in addition to presenting long-term relationships between the model variables.

**Table 3:** Short-run estimates.

Variable	Coefficient	Std. Error	t-Statistic	Prob.*
LRGDP(-1)	0.153685	0.206252	0.745133	0.4670
LNINF	0.021529	0.026181	0.822343	0.4230
LNINF(-1)	0.046804	0.031373	1.491858	0.1552
LNINF(-2)	0.053851	0.026948	1.998377	0.0630*
LUN	−0.249041	0.091507	−2.721552	0.0151**
C	7.458776	1.940411	3.843916	0.0014***
@TREND	0.054667	0.011782	4.639893	0.0003***
R^2^	0.984694		Mean dependent var	9.014429
Adj R^2^	0.978954		S.D. dependent var	0.334612
S.E. of regression	0.048543		Akaike info criterion	−2.966933
Sum squared resid	0.037703		Schwarz criterion	−2.621347
Log likelihood	41.11973		Hannan-Quinn criter.	−2.880019
F	171.5531		Durbin-Watson stat	1.876346
P (F)	0.000000			

Note: *, ** and *** denote stationary at 10 %, 5 % and 1%significance levels.

In order to find the best numbering of time periods for variables given their limited study period (1991–2020), the researchers used a number of factors. Additionally, the Schwarz-Bayesian criterion was used to make the decision. Only one delay was returned to the LnRGDP variable, two delays were returned to the LnINF variable, and LnUN received no delays in this model. The outcomes for F and R2 show that the data collected for looking at the variables is considered reliable and accurate. The selection was made using the Schwarz-Bayesian criterion. However, the researchers utilized a number of factors to identify the optimal numbers for the time periods of the variables due to its limited investigation, which was between these years (1991–2020). The LnINF variable received two delays, LnUN received none, and just one LnRGDP variable was returned as a delay and considered as a result. While the short-term outcomes of the distributed time gap model revealed that there is no significant influence for the first period of inflation, the F and R2 statistics findings demonstrate that the data gathered for studying the variables are sound and accurate. Although the results are encouraging, it was discovered that the second period of inflation has a substantial impact at the level of a substantial 10%. This is because an increase in inflation rates of 1% translates into a short-term increase in real GDP of (0.053851). This is so that market mechanisms can move, processes of supply and demand can be balanced, and economic stability may be maintained. It will also enable prices to adapt and return to their pre-crisis form. In the case of the Palestinian economy, inflation will contribute to rapid growth in the short run. These results are consistent with [13], who found that there is a causality between GDP and inflation in an agreed context [14]. found out that when inflation rises by 0.906 %, the GDP will also rise by 1 %. These results differed from the study of [15], which discovered a negative effect of inflation on the gross domestic product. The results explain a negative significant effect of unemployment at the level of 1 % without any delay. As if unemployment increased by 1 %, the real GDP would decrease by (2.49041). The researchers attribute this result to the lack of job opportunities due to the high population growth, especially in the Gaza Strip. It is a narrow geographical space and a high population, and lack of alignment of scientific disciplines with market requirements. In addition to the inferior view of vocational and technical education, which makes unemployment is widespread in the short term in a large way. This is consistent with the [17] study, which showed a significant negative impact on economic growth on unemployment and the [19] study which shows a negative effect of unemployment on economic growth in the short run.

d. **(ARDL)** Model in long-period

Table 4, ARDL was used to evaluate the tall-run growth model coefficients based on the Schwazer-Bayesian criterion.

**Table 4:** Long-run estimates.

V	Coeff	Std. Err	t	P
LNINF	.144372	.082171	1.756984	.0980*
LUN	−.294265	.066486	−4.425967	.0004***
C	8.813241	.266309	33.094036	.0000***
@TREND	.064595	.003871	16.686298	.0000***

The study's findings demonstrate that all factors are consistent with the hypothesis' basic premises since they reveal that inflation has a long-term positive and significant impact on projected real GDP at a considerable level of 10%. Furthermore, a rise in economic growth of 0.1443 % results from a 1% increase in inflation. This implies that inflation is a crucial element in establishing economic growth in Palestine. It also creates a healthy work environment, as the high operating rate of prices helps the current merchants to continue their work by achieving a distinct profit margin without worrying about the idea of falling prices and making losses. Consequently, inflation increases economic growth because of the entry of Arab citizens of Israel to buy from the Palestinian Territories, which improves the economic situation. The results of this hypothesis are consistent with the study of each of [19]. The results of this study differed from those [17] who found a Negative effect of inflation on economic growth.

Similarly, assuming that other factors remain constant, it has been observed that a one per cent point increase in unemployment in Palestine leads to a decrease in long-run economic growth by 0.294265%. This result is consistent with Okun's Law, which shows that unemployment and Palestinian economic growth are inversely correlated. Palestine has high unemployment rates, which hints at a lack of full utilization of human capital in the country, which leads workers to lose their income. In fact, Palestine is suffering from numerous problems such as continuous wars and repeated security campaigns, which make Palestine an inappropriate environment for long-term investment, which keeps investors away from this and increases the unemployment rate. This result is supported by the research [3,15], while these results differed from the study of [18], which was not related to unemployment on economic growth.

e The Error-Correction Model (ECM)

Since the (ECM) model is used to connect short-lived fluctuations with long-lived value, co-integration of macroeconomic variables is a requirement rule so that the researchers can use it. Additionally, as indicated in Table 5 below, the researchers can modify the (ECM) using the value residuals of the Least Squares Model (OLS).

**Table 5:** ECM estimates.

V	Coefficient	St. Err	t	P*
D(LNINF)	−.019773	.023302	−.848539	.4095
D(LUN)	−.169566	.082415	−2.057470	.0575*
ECM(-1)	.018381	.087579	.209877	.8366
C	.056729	.042515	1.334320	.2020
@TREND	.000238	.002436	0.097726	.9234
R^2^	.615786		M d var	0.047110
Adj R^2^	.462100		S.D. d var	0.080455
S.E. of regression	.059007		Akaike info criterion	−2.568939
Sum squared resid	.052228		Schwarz criterion	−2.221790
Log likelihood	35.25833		Hannan-Quinn criter.	−2.487161
F	4.006787		DW	2.782858
P (F)	.013564			

The findings of the study demonstrate that the principles and metrics applied to the proposed theory were inconsistent with the study. In relation to this context, the inflation variable was compatible with the error correction approach, making it unimportant. However, it is considered to be negative, which indicates that inflation, if it exceeds the normal limit, will negatively affect the Palestinian economy, and therefore; it is necessary to follow methods that stimulate healthy inflation within the market. While the unemployment variable has a statistically significant negative sign at the level of 10%. This shows that a 1 % increase in unemployment causes a 0.1695 % short-term drop in economic growth, For this reason, the government should invest in human capital in a healthy manner by paying to choose work specializations that meet the needs of the Palestinian market.

The previous Table 4 makes it clear that the value of F is equal to 4.006. This value verifies that all examined coefficients are significant and correct. In contrast, the ECM coefficient [ecm (−1)] was positive and equal to (0.0183) and considered not significant. These results demonstrate the absence of specific government mechanisms to lead economic growth towards balance quickly because the Palestinian government does not possess many tools for fiscal and monetary policies. Moreover, the fact that the currencies are foreign currencies such as the Israeli shekel, the Jordanian dinar, and the US dollar makes it difficult to follow monetary policies related to inflation except for the high interest rate because it does not deal with bonds and the discount rates greatly. Nevertheless, it is outside authority due to religious considerations, since bank interest is forbidden according to Islamic law. Therefore, in cases of inflation, if any, there is no ability to solve this crisis, especially after the repercussions of the COVID-19 pandemic and its consequences. For this reason, It is difficult to carry out real development operations inside Palestine to reduce unemployment due to the arbitrary policies of the Israeli occupation. Achieving an economic renaissance is difficult due to the loss of local capital and the difficulty of pumping foreign investment because of deprivation, which leads to an exacerbation of the situation.

f. CUSUMSQ and CUSUM *t*-Test Results

According to Ref. [25], After calculating the ECMARDL formula, the next step focuses on looking at the short- and long-term structural stability coefficients. This indicates that there haven't been any significant changes over time to the data utilized in this study. Both the cumulative sum of squares test (CUSUMSQ) and the cumulative residuals (CUSUM) are employed. It should be mentioned that the graph of the statistics of CUSUM and CUSUMSQ must be centered within the critical limits at a substantial level in order for the ARDL model to be structurally stable. The results of the stability test are shown in the following figure. The results of Test No. 03, which examined the structural stability of the estimated ARDL model, are displayed.

**Fig. 1:**
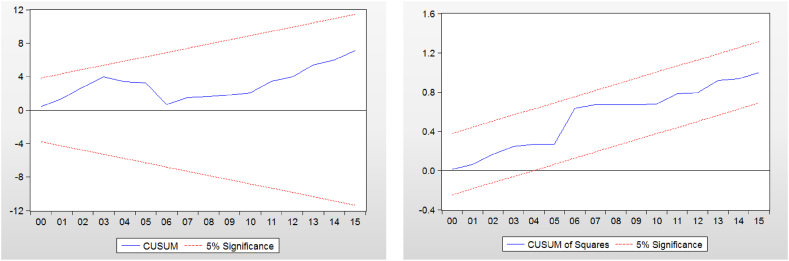
Results of the calculated ARDL model's structural stability test.

Fig. 1 makes it obvious that the predicted coefficients for the employed ARDL model are stable. Additionally, it should be noted that the cumulative sum of squares (CUSUM of Squares) and the sum of the CUSUM are both median lines that are almost exactly within the boundaries of the critical region, which suggests that the ARDL model is structurally stable at the level of 5% significance for the selection of (CUSUM of Squares) are a median line located almost within the boundaries of the critical region. This means that the structural stability of the ARDL model is at the level of 5% significance for the choice of (CUSUM of). The CUSUM Test shows that the parameters are stable at a significant level of 5%, confirming that the research variables are stable and that the model's short-term and long-term results of error correction are in agreement with one another. Hence, the critical limits are at the 5% level of importance.

## 6 Conclusion

This study focuses on numerous difficulties that developing nations like Palestine must overcome to attain steady and long-term economic growth. Studying how unemployment and inflation factors can affect economic growth, particularly in Palestine. The study also aims to determine whether there is a long-term relationship between these two factors. Time series data from 1991 to 2020 are used in the study, and ARDL and ECM estimation methods are used for analysis. According to the findings, both long- and short-term economic growth in Palestine is significantly hampered by unemployment. However, unlike the anticipated pattern, there was no evidence of a short-term impact of inflation on economic growth, even if inflation ultimately had a very positive impact on it. In contrast to unemployment, which was determined to be statistically significant at 10 %, the data showed that the error-corrected term (ECT) and inflation were not statistically significant. The vast living disparity between the two regions of the country, the West Bank and Gaza, which creates an opposite living gap between them, was one of the real determinants that this study had to contend with. In the West Bank, unemployment is low and inflation is high, while, in Gaza, inflation is low and unemployment is high. Gaza Strip suffered from five devastating wars within a decade, which made it difficult for researchers to collect data, some of which were corrected after submission. The Palestinian Central Bureau of Statistics indicated that it was received from the World Bank.

This research provides policy recommendations for Palestine based on the generated findings. This indicates that effective control of market prices or stabilization of inflation through effective price control mechanisms can enhance macroeconomic conditions, increase investor confidence and stimulate economic growth so that in the end more output can be produced at a lower cost per unit, thus achieving sustainability and economic growth. Eliminating unemployment requires creating opportunities for the full use of human resources by stimulating self-employment to engage in entrepreneurial activities according to the qualifications of graduates, providing the population, especially youth, with relevant skills to increase productivity and create orientation. This means that students need vocational education, which is important in the labor market. In addition, it is recommended to improve institutional quality, strengthen national unity, review trade policies, and reduce the possible negative short-term effects of the conflict in the Gaza Strip on economic growth. However, more importantly, this will be crucial in ensuring Palestine's sustainable economic growth and its factors such as; labor, force, technology and land.

## Funding statement

No funding was received for this work.

## Data availability statement

The authors do not have permission to share data.

## Additional information

No additional information is available for this paper.

## CRediT authorship contribution statement

**Alaa Razia:** Investigation, Supervision, Writing - original draft. **Mostafa Omarya:** Methodology, Resources, Writing – original draft. **Bahaa Razia:** Software, Writing - review & editing. **Bahaa Awwad:** Formal analysis, Conceptualization, Data curation. **Abdullah Ruzieh:** Project administration, Validation, Visualization.

## Declaration of competing interest

The authors declare that they have no known competing financial interests or personal relationships that could have appeared to influence the work reported in this paper.
